# Knowledge, attitudes, practices, and future intentions to use intermittent preventive treatment with sulfadoxine-pyrimethamine among pregnant women in southern Ghana

**DOI:** 10.1186/s12884-026-09063-8

**Published:** 2026-04-15

**Authors:** Matilda Aberese-Ako, Wisdom Ebelin, Pascal Magnussen, Kingsford Norshie, Desmond Klu, Mustapha Immurana, Gifty D. Ampofo, Harry Tagbor

**Affiliations:** 1 Institute of Health Research, University of Health and Allied Sciences, PMB 31, Ho, Volta Region Ghana; 2 Evangelical Presbyterian Health Services, Evangelical Presbyterian Headquarters, Ho, Volta Region Ghana; 3 Dept. of Immunology and Microbiology, Centre for translational Medicine and Parasitology, University of Copenhagen, Copenhagen, Denmark; 4 School of Medicine, University of Health and Allied Sciences, Ho, Volta Region Ghana

**Keywords:** Malaria, IPTp-SP, Pregnant women, Ghana, Healthcare providers, Qualitative study, Knowledge, Attitudes, Practices, Antenatal care, Health belief theory

## Abstract

**Introduction:**

Malaria in pregnancy is a significant public health concern in sub-Saharan Africa, affecting an estimated 32 million pregnant women annually. One of WHO’s recommendations for malaria prevention is the use of intermittent preventive treatment in pregnancy with sulfadoxine-pyrimethamine (IPTp-SP). However, reports indicate that less than 100% of women in Ghana access IPTp service. This study explored knowledge, attitudes, uptake, and future intentions to take IPTp-SP among pregnant women in southern Ghana.

**Methodology:**

An ethnographic study design was used. Data was collected from April 20,218 to March 2019 using in-depth interviews, conversations, and non-participant observations, among pregnant women, health workers, managers, and a cross-section of community members. The interviews were recorded digitally, transcribed, and uploaded together with the transcribed conversations and observation notes into NVivo Version 11 for triangulation, coding, and thematic analysis. All ethical procedures were followed.

**Results:**

Six major themes were identified: The context of SP administration as observed at the ANC, Post-SP advice and counseling by health providers, Knowledge of the benefits of intermittent preventive therapy in pregnancy, Frequency of SP uptake and Knowledge of recommended dosing, experience in taking in SP, and intentions of future uptake of SP. The findings show that SP uptake was well integrated into routine antenatal care and delivered through a structured, provider-led process under direct observation. Mandatory water intake and advice to eat heavy meals were common practices. Counselling on SP varied across clients and was generally limited after administration, particularly regarding side effects. Most participants received three or more SP doses in line with national guidelines and demonstrated strong trust in healthcare providers. However, knowledge of correct dosage and timing was limited, with misconceptions evident. Despite experiencing discomfort such as nausea and dizziness, most women continued SP use due to perceived maternal and fetal benefits.

**Conclusion:**

Strengthening communication between healthcare providers and pregnant women regarding dosing schedules, potential side effects, and follow-up doses could improve adherence and optimize the effectiveness of malaria prevention strategies.

**Supplementary Information:**

The online version contains supplementary material available at 10.1186/s12884-026-09063-8.

## Background

Malaria in pregnancy remains a major public health challenge, particularly in sub-Saharan Africa. In 2022, the WHO African Region reported approximately 233 million malaria cases, accounting for 94% of global infections [1]. That same year, West Africa recorded the highest prevalence of malaria exposure among pregnant women, with about 6.4 million (39.3%) out of an estimated 16.2 million pregnant women affected [2].

To mitigate the impact of malaria in pregnancy, the World Health Organization (WHO) recommends several preventive strategies for women living in areas with moderate to high malaria transmission in Africa [1]. These include the use of long-lasting insecticidal nets (LLINs), effective case management through prompt diagnosis and treatment, and intermittent preventive treatment in pregnancy with sulfadoxine-pyrimethamine (IPTp-SP) administered under directly observed therapy (DOT) during antenatal care (ANC) visits [3, 4]. IPTp-SP involves administering a full therapeutic dose of sulfadoxine-pyrimethamine to pregnant women at each scheduled ANC visit, starting in the second trimester, with doses administered at least one month apart [1].

In Ghana, malaria continues to be a leading cause of morbidity and mortality. In 2017, malaria accounted for 34% of all outpatient department (OPD) cases, 19% of hospital admissions, and 2% of total deaths [5]. That same year, 399,736 suspected cases of malaria in pregnancy were recorded, of which 133,687 (33.4%) were confirmed [5]. Malaria infection during pregnancy contributes significantly to maternal and fetal complications, including illness and death [6]. Although Ghana has achieved considerable progress in reducing malaria incidence, a substantial burden persists among pregnant women [7].

To enhance malaria prevention, Ghana revised its IPTp policy, shifting from a fixed three-dose regimen to a flexible schedule allowing a minimum of three and up to seven doses of SP, administered monthly from the second trimester until delivery [8]. This intervention is integrated into the country’s free maternal health policy through ANC services [9]. However, as of 2023, only about 78% and 60% of pregnant women had received at least two and three doses of SP/Fansidar, respectively, figures that fall below the National Malaria Elimination Programme’s target of 85% established in 2011 [10, 11].

Despite these efforts, several critical questions remain: Why are the national coverage targets not being achieved? How do pregnant women perceive SP? What are their experiences and attitudes toward taking SP, and what influences their future intentions regarding its uptake? Previous studies have attempted to address these questions [12–17]. Nationally, only 30.5% of pregnant women received the recommended ≥ 3 doses of IPTp-SP, with women aged 15–24 years significantly less likely to achieve optimal uptake compared with those aged 35–39 years (aOR = 0.25, 95% CI: 0.09–0.71) [13]. Health service factors consistently influenced uptake. In the Ashanti Region, late antenatal care (ANC) initiation (AOR = 0.4, 95% CI: 0.21–0.89; *p* = 0.022) and inappropriate timing of IPTp-SP administration (AOR = 0.1, 95% CI: 0.03–0.37; *p* < 0.001) were negatively associated with receiving ≥ 3 doses, while good knowledge (AOR = 6.5, 95% CI: 1.06–39.72; *p* = 0.043) and perceived therapeutic effectiveness (AOR = 3.4, 95% CI: 1.08–11.0; *p* = 0.037) were positive predictors [15]. Similarly, attending ≤ 3 ANC visits markedly reduced the likelihood of optimal uptake (AOR = 0.03, 95% CI: 0.01–0.83; *p* < 0.001).In the Volta Region, adequate uptake (3–4 doses) was reported among 64.6% of women despite poor perception (63.4%), although awareness was high (87.5%) [14]. Knowledge (45.9%) and attitude (58.9%) levels were moderate in related district-level findings [16]. In the Ahafo Region, higher uptake was associated with perceived severity of malaria in pregnancy (AOR = 0.19, 95% CI: 1.02–4.20; *p* = 0.045) and perceived benefits of IPTp-SP (AOR = 0.39, 95% CI: 0.19–0.78; *p* = 0.008), while perceived susceptibility and sociodemographic factors were not significant predictors [17].

This qualitative study seeks to build upon existing literature by exploring the knowledge, attitudes, uptake, and future intentions regarding the use of SP among pregnant women in southern Ghana. The findings aim to provide deeper insights into contextual and behavioral factors that influence malaria prevention practices during pregnancy.

### Theoretical framework guiding the study

This study is guided by the Health Belief Model (HBM), which posits that individuals’ engagement in health-related behaviours is shaped by their perceptions of disease risk and the anticipated consequences of illness [18–20]. HBM has three constructs: perceived susceptibility, perceived severity, and perceived benefits and barriers, which together provide a useful framework for understanding how beliefs influence decision-making regarding the uptake of preventive health interventions.

The first construct, perceived susceptibility, refers to individuals’ beliefs about their personal likelihood of experiencing a disease. These perceptions vary widely, ranging from a belief that one is not at risk to a heightened sense of vulnerability. In the context of this study, perceived susceptibility relates to how pregnant women understand and assess their risk of malaria during pregnancy and how this perception influences their willingness to accept intermittent preventive treatment in pregnancy with sulfadoxine–pyrimethamine (IPTp-SP).

The second construct, perceived severity, concerns beliefs about the seriousness of a disease and its potential consequences. This includes not only clinical outcomes, such as illness or complications, but also broader social, economic, and personal implications. In this study, perceived severity captures women’s understanding of the potential effects of malaria on maternal and fetal health.

The third construct combines perceived benefits and perceived barriers, reflecting individuals’ evaluation of the advantages of taking a health-related action in relation to the obstacles that may hinder it. Perceived benefits may include protection against malaria and improved pregnancy outcomes, while perceived barriers may involve concerns about side effects, cost, accessibility, or the quality of interactions with health providers. High perceived benefits are expected to motivate uptake of IPTp-SP, whereas significant barriers may discourage its use [18–20].

The HBM has been widely applied in health research to examine behaviours related to access to healthcare services, preventive interventions, and treatment adherence [21–23]. In this study, the model informed the adoption of an ethnographic design, enabling prolonged engagement in eight antenatal care (ANC) clinics and eight communities. This approach facilitated in-depth exploration of interactions between health providers and pregnant women and how these interactions shape knowledge, attitudes, uptake, and future intentions regarding IPTp-SP among women in southern Ghana.

## Methods

### Study design

An ethnographic descriptive study design was used. The approach enabled the study to explore how routine interactions between health providers and ANC clients in the health care environment shape women’s experiences in IPTp-SP uptake in Ghana. The health belief model offered a theoretical framework to explain decision on SP uptake based on pregnant women’s perceptions of susceptibility to and severity regarding SP uptake in a malaria-endemic country.

The study employed non-participant observations, case studies, informal conversations, and in-depth interviews (IDIs). In-depth interviews (IDIs) were conducted using semi-structured interview guides to gather information from healthcare providers, healthcare managers, pregnant women (majority of them were ANC registrants), and community members.

The team used informal conversations, defined as “an unplanned and unanticipated interaction between an interviewer and a respondent that occurs naturally during the course of fieldwork observation” [24], to gather information from women attending ANC and health providers. In contrast, IDIs were more formal, as research assistants used semi-structured interview guides containing probes, transitions, and follow-up questions. These guides offered greater structure, direction, and control, and facilitated more detailed data collection than the informal conversations [25],

Data was collected between April 2018 to March 2019. The research team comprised of a female medical anthropologist (MA) and nine graduate research assistants (RAs). Of the RAs, three were female, and six were male, all of whom spoke the indigenous language of their assigned study areas: the Twi language for RAs recruited in the Ashanti Region and the Ewe language for those recruited in the Volta Region. The RAs underwent training on how to carry out community entry, IDI procedures, informal conversations, observational techniques, and writing field notes in line with the guidelines of Emerson and Fretz [26], prior to and during data collection.

The RAs observed and documented antenatal care (ANC) service provision across eight study facilities and eight communities. To minimize the Hawthorne effect, observations were conducted intermittently in all facilities and communities [27].

### Selection of research area

The study was conducted in five districts, three in the Ashanti region and two in the Volta region of Ghana, chosen to represent the country’s middle and southern belts, respectively, with distinct languages (Twi in Ashanti and Ewe in Volta) [18, 19]. Eight health facilities and eight communities were selected, including five district hospitals and three faith-based facilities preferred by women due to their proximity (Table 1: indicates the number of facilities and communities per region using coded names). These preferences were confirmed through interviews and a transect walk in the study communities to identify key places, settlement patterns and physical access to health care facilities and other sources of health care. The study reviewed antenatal care (ANC) and maternity admission records for malaria in pregnancy (MiP) cases from January 2015 to March 2018, selecting one community per facility with the highest number of MiP cases, each averaging 10,000 inhabitants. Community entry activities were conducted to inform and gain approval from local leaders.

**Table 1:** Study health facilities and communities in the Ashanti and Volta Regions with pseudonyms

Type of study site	Region	
	**Ashanti** ^a^ No.	**Volta** ^b^ No
Hospital(s)	3	2
Health Centre(s)	1	2
Ownership of facility		
Government owned	2	3
Mission owned	2	1
Communities	4	4

^a﻿^Study facilities in the Ashanti region have been given the following pseudonyms: ASF01, ASF02, ASF03, and ASF04. Study communities in the Ashanti region have been given the following pseudonyms: ASC01, ASC02, ASC03, and ASC04

^b﻿^Study facilities in the Volta region have been given the following pseudonyms: VRF01, VRF02, VRF03, and VRF04. Study communities in the Volta Region have been given the following pseudonyms: VRC01, VRC02, VRC03 and VRC04.

Source: Aberese-Ako et.al. [28]

### Selection and recruitment of study participants

Each research assistant (RA) was assigned to a health facility to interact with healthcare providers and pregnant women attending ANC. Using convenience sampling, pregnant women willing to participate were recruited, and their phone numbers were recorded for follow-up interviews at locations of their choice [28]. Also, snowball sampling was employed to recruit some of the pregnant women from the eight study communities [29]. The initially recruited participants at the health facilities assisted research assistants in identifying other pregnant women within their communities. So, the RAs followed up to the communities to invite such women to participate in the study. The study was explained to all potential participants, and those who agreed to participate were enrolled in IDIs after providing written informed consent. All pregnant women who attended ANC during the twelve-months period of data collection were included in the study. Additionally, pregnant women who were not attending ANC in the study communities were included. Another inclusion criteria were the participants’ ability to communicate in English or in the indigenous language. All pregnant women who declined to participate in the study were excluded.

In addition, community opinion leaders, including assembly members, mothers, and mothers-in-law of pregnant women, were invited to participate in IDIs.

Case studies were purposively selected from women who attended ANC monthly, as well as those who attended irregularly or who missed ANC appointments. A total of twelve case studies were followed throughout the study period (Table 2). These women were visited several times in their homes, where research assistants observed medication-taking practices, adherence to ANC appointments, experiences from previous ANC visits; particularly regarding the provision of sulphadoxine–pyrimethamine (SP) and the use of long-lasting insecticidal nets (LLINs). Maternity record booklets were also reviewed to verify the information provided.

Healthcare providers, mainly midwives and nurses delivering ANC services, with at least one year of work experience in a health facility, were recruited to participate in the study. ANC unit managers (commonly referred to as “in-charges”) and facility managers, including senior medical officers, physician assistants, and administrators, were interviewed to gain insight into managerial and administrative processes. The study team also conducted follow-up informal conversations and interviews with procurement officers, laboratory personnel, and officials at the district health directorate to clarify issues emerging from earlier IDIs and discussions with healthcare providers and managers. Details of the various categories of study participants and the data collection methods employed are presented in Table 2.

**Table 2:** Data collection methods and categories of respondents

Region	Category of Respondents	IDIs	Conversations	Case studies	ANC interactions^d^
Ashanti^a^	Health Managers	8	4	0	-
	HealthCare providers	11	20	0	-
	Pregnant women	30	25	4	40
	Opinion Leaders	10	5	0	-
	Procurement officers	1	2	0	-
	Laboratory officials	0	2	0	-
	DHD^b^ officials	**0**	**2**	**0**	**-**
	Total	**60**	**60**	**4**	**40**
Volta^c^					
	Health Managers	8	4	0	-
	HealthCare providers	12	20	0	-
	Pregnant women	40	32	8	40
	Opinion Leaders	14	6	0	-
	DHD Officials	0	2	0	-
	Total	**74**	**64**	**8**	**40**

^a^Observations were carried out in 4 health facilities and 4 communities from April, 2018 to March 2019 in the Ashanti Region

^b^District Health Directorate

^c^Observations were carried out in 4 health facilities and 4 communities from April 2018 to March 2019 in the Volta Region

^d^Eighty ANC interactions between health providers and clients were observed: 40 in the Ashanti region and 40 in the Volta region. An average of 10 were observed in each of the four facilities in each region

Source: Aberese-Ako et. al. [28]

### Data collection techniques and process

A team of trained research assistants (RAs) conducted prolonged non-participant observations over several months in each selected health facility to document antenatal care (ANC) service delivery and interactions between healthcare providers and pregnant women. Prolonged engagement in the field enhanced contextual understanding and strengthened the credibility of the findings. Initial observations focused on core ANC activities, including client registration, health education sessions, blood pressure and urine protein measurements, consultations in ANC rooms, administration of sulphadoxine–pyrimethamine (SP), and visits to laboratory and pharmacy units.

To capture the continuity of care and gain insight into women’s lived experiences within the ANC pathway, RAs randomly selected ANC attendees and accompanied them throughout their service trajectory. Permission was obtained from healthcare providers, and informed verbal consent was secured from the women prior to participation. During these observations, RAs engaged women in informal conversations to clarify observed procedures and actions, thereby enhancing interpretive validity. Detailed field notes were recorded contemporaneously and later transcribed verbatim to ensure dependability and facilitate systematic analysis.

Observations and discussions with pregnant women explored their knowledge of SP, understanding of malaria in pregnancy (MiP), and intentions regarding SP uptake. Engagements with healthcare providers focused on SP-related policies, drug availability, counselling practices, and information provided before and after SP administration. The use of multiple data sources such as direct observations, informal conversations, and interviews enabled methodological triangulation and strengthened the credibility of the findings.

In-depth interviews (IDIs) and informal conversations with pregnant women and community members were conducted in local languages (Ewe in the Volta Region and Twi in the Ashanti Region) to ensure cultural sensitivity and promote authentic expression. These interviews explored knowledge, attitudes, beliefs, practices, and socio-cultural factors influencing MiP interventions. To enhance credibility and confirmability, SP obtained from the facilities was shown to participants during interviews, and women were asked to identify and describe the correct dosage (three tablets per administration). This strategy served as a form of member verification and reduced recall bias.

IDIs with healthcare providers, healthcare managers, and National Health Insurance Scheme (NHIS) officials were conducted in English, Ghana’s official language. These interviews examined maternal health and MiP policies, service delivery processes, implementation challenges, and facilitating factors. With participants’ consent, maternity record booklets were reviewed to verify documented uptake of intermittent preventive treatment with SP, thereby supporting data triangulation and enhancing confirmability.

To further strengthen trustworthiness, the lead investigator (MA) conducted monthly supervisory visits to the study sites and periodically participated in data collection alongside the RAs. Weekly debriefing meetings (through conference calls using mobile phones) were held with all nine RAs to review emerging findings, address methodological challenges, ensure adherence to study protocols, and promote reflexivity. These ongoing discussions supported analytic rigor and consistency in data collection.

The study instruments were developed using constructs from the Health Belief Model including knowledge, attitudes, perceived benefits and barriers, practices, and future intentions. To enhance validity and cultural appropriateness, interview guides and observation checklists were pretested in English, Ewe and Twi within communities with similar characteristics to the study sites. Feedback from the pretesting informed refinement of the tools. Additionally, refresher training sessions were conducted during supervisory visits to strengthen RAs’ ethnographic competencies and ensure consistency in observation and interviewing techniques, thereby enhancing dependability.

### Data analysis

All interviews were digitally recorded and transcribed verbatim to preserve participants’ original meanings. Interviews conducted in Ewe and Twi were translated into English to facilitate systematic analysis and comparison across data sources. Analytical rigor was enhanced through prolonged engagement with the data; MA iteratively reviewed in-depth interviews (IDIs), observation notes, and informal conversations throughout the fieldwork period to familiarize herself with emerging themes and sub-themes. Identified data gaps and emerging insights informed follow-up activities conducted by research assistants (RAs), and the resulting information was incorporated into the dataset. Triangulation was achieved by integrating multiple data sources (IDIs, observations, and conversations) and involving multiple analysts in the coding process. Coding, analysis, and data collection proceeded concurrently until data saturation was reached, defined as the point at which no new information emerged regarding the major themes, consistent with recommended qualitative standards [30].

A systematic and transparent analytic process was maintained to enhance dependability. Revised transcripts, observation notes, and conversation records were labelled, finalized, and uploaded on a rolling basis into NVivo version 11 to support organized coding and data management. MA and ED, a qualitative data analysis expert, jointly developed a codebook based on the IDI guide, conversation checklist, and initial observation notes. The coding process followed an iterative approach combining deductive and inductive strategies, with continuous refinement of themes and sub-themes. Coding was conducted over a five-month period, during which datasets were regularly compared, discrepancies discussed, and relevant sections re-coded to ensure consistency.

Confirmability was strengthened through investigator triangulation and external validation. In addition to MA and ED’s collaborative coding, a third coder (WE) independently reviewed and validated the coded data through systematic examination. Following consensus on the themes and sub-themes, WE transferred the coded data into a matrix to support further structuring and interpretation [9]. This multi-coder approach helped minimize individual researcher bias and ensured that findings were grounded in the data.

The analysis focused on identifying similarities, patterns, differences, and contradictions across the triangulated data sources. The resulting themes and sub-themes (Table 3) formed the basis for the interpretation and reporting of findings [31]. The study explored health system, socio-cultural, and community-level factors influencing the utilization of malaria in pregnancy interventions within Ghanaian communities. While the findings are context-specific, the detailed description of methods and analytic processes enhances the potential transferability of insights to similar settings. Components of this broader research agenda have been reported elsewhere [28].

**Table 3:** Major and sub-themes derived from the analysis of the data

Major themes	Sub-themes
The Context of SP Administration as observed at the ANC	• Integration of SP with routine ANC services • Structured and provider-led process of SP uptake under direct observation • Mandatory water intake • Advice to eat heavy meals before uptake • Variable levels of counselling depending on client history and clinical presentation at the facility • Provision of information on SP and LLINS • Laboratory test required for clients with complaints akin to malaria infection prior to SP uptake
Post-SP Advice and Counseling by health providers	• Limited post-administration information • Rare Post SP Counseling • Limited advice on managing side effects • Selective and inconsistent health guidance
Knowledge of the benefits of intermittent preventive therapy in pregnancy	• Benefits to mother • Benefits to the unborn child • Effectiveness of SP limits the use of other preventive methods, such as ITN • Misconceptions regarding IPTp-SP
Frequency of SP uptake and Knowledge of Recommended Dosing	• Limited knowledge of recommended SP dosage • Reliance on health providers’ authority • Partial or incorrect information on the recommended number of doses • Changes in SP dosage practices over time
Experience in taking in SP	• Negative experiences and physical discomfort associated with SP intake • Pregnancy-related appetite issues make SP pre-heavy food intake a challenge • Absence of adverse reactions to SP intake
Intentions of future uptake of SP	• Acceptance and continued willingness to use SP despite discomfort • Conditional acceptance and discontinuation of SP use

### Ethical issues

Ethical clearance for the study was obtained from the University of Health and Allied Sciences’ Research Ethics Committee (UHAS-REC A.1 [l] 17–18), and written informed consent was secured from all interview participants or their legal guardians. Women attending ANC who expressed interest in the study were given time to consider participation. Those who remained interested were contacted later by research assistants and provided with consent forms before the interviews; one 16-year-old participant was included with her mother’s permission. Approval to conduct the study was also obtained from relevant district health authorities, facility managers, community leaders, and chiefs. To ensure confidentiality, pseudonyms were used for districts, individuals, and facilities. For the Ashanti Region, ASF and ASC were used for facilities and communities, while VRF and VRC were used for those in the Volta Region. Respondent names are pseudonyms based on the community where the interview took place.

### Findings

The findings present the background characteristics of the women who were interviewed, observed in the ANC, as well as those we held conversations with, the context of the ANC and SP administration, women’s interaction with health providers at the ANC regarding SP uptake, experience in SP uptake, and future intentions of uptake the ANC.

### Background characteristics of study participants

The ages of the pregnant women who participated in the IDIs, conversations, and those recruited for the case study ranged from 16 to 38 years, with the majority in their early to mid-twenties and early thirties. Educational attainment was generally low to moderate, with most participants having completed basic or junior high school education. A smaller proportion had senior high school education, while only a few attained tertiary or vocational education, and some reported no formal education. This educational distribution reflects the broader educational context of women in the study communities and provides important context for understanding variations in knowledge and perceptions observed in the findings. In terms of occupation, participants were predominantly engaged in informal economic activities, including trading, seamstress work, hairdressing, farming, food vending, and small-scale businesses. A notable number were unemployed, particularly among those who were below age 24. Parity varied across participants. While some women were nulliparous, many had one to four children, indicating a mix of first-time and experienced mothers.

### The context of SP administration as observed at the ANC

Overall, SP delivery was characterized by strict adherence to DOT, prioritization of provider authority, and high levels of compliance, while counselling practices, access to water, and facility resources varied and influenced implementation.

Women were called individually into the consulting rooms on a first-come, first-served basis. Each woman was welcomed by the health provider, offered a seat, and engaged in a brief conversation before undergoing a physical examination from head to toe to identify any signs of conditions that could affect the health of the mother or the unborn child. The woman’s ANC booklet was reviewed, and if she was due for intermittent preventive treatment with sulfadoxine-pyrimethamine (SP) (observation notes from eight facilities: ASFacility01, ASFacility02, ASFacility03, ASFacility04, VRFacility01, VRFacility02, VRFacility03 and VRFacility04).

Across all eight health facilities, antenatal care (ANC) observations showed that sulphadoxine–pyrimethamine (SP) delivery followed a highly structured, provider-led process embedded within an integrated routine ANC service delivery. SP administration was consistently conducted under directly observed therapy (DOT), with nurses or midwives exercising strong authority to ensure compliance. Women were called individually, and SP was administered in designated consulting rooms as part of integrated ANC procedures that included clinical assessment, palpation, dietary counselling, and review of prior health conditions (observation notes from: ASFacility01, ASFacility02, ASFacility03, ASFacility04, VRFacility01, VRFacility02, VRFacility03 and VRFacility04). In ASFacility01 in the Ashanti Region SP was taken in the OPD space (ASFacility01, observation note FN001). In another facility in the Volta region women were called individual typically through a public address system (VRFacility03, observation note FN003).

Prior to SP administration, providers routinely assessed whether women had eaten and had access to water. Women who reported not having eaten, or who had consumed what providers deemed a “light” meal (e.g., porridge or tea), were instructed to eat a heavier meal before returning to take SP. Similarly, women without water were required to purchase it before administration. In facilities where water was not provided, women frequently left the ANC area to obtain it. Only VRFacility02 in the Volta region provided water to women to take in SP. In some instances, women requesting to take SP outside the facility, such as while fetching water, or to take it home, as some complained of dizziness if they took in SP, were refused, and were instead required to leave their ANC booklet as collateral and return to swallow the tablets under supervision.

DOT enforcement was uniform, although variation was observed in how women ingested the tablets, including chewing them, swallowing them one at a time, or swallowing all three at once. Regardless of method, providers ensured ingestion occurred in their presence. Conversations with providers revealed that refusal of SP was rare (VRFacility02, conversation with a health provider). When women expressed reluctance or discomfort, nurses actively encouraged and persuaded them to comply, reflecting both high acceptance of SP and strong provider enforcement of facility protocols. A nurse in one facility explained that if a woman in the process of swallowing SP vomited, they went through the vomit to determine whether the SP tablets were in it, and if it was, the woman would be required to take the lost tablet ASFacility02 Sup001, conversation with a health provider).

Health education related to SP was uneven across facilities. First-time recipients were more likely to receive counselling on the purpose of SP, its timing from the fourth month of pregnancy, and its role in malaria prevention alongside insecticide-treated bed nets. Women receiving SP for the first time were also issued bed nets. However, counselling was less consistent for repeat recipients.

Women presenting with malaria-related complaints or a history of malaria were sometimes referred for laboratory investigations prior to SP administration, and those identified as Glucose-6-phosphate dehydrogenase (G6PD) deficient were excluded from receiving SP. (ASFacility02, observation notes. SUP002, VRFacility02, observation note FN001)

### Administration of SP during antenatal care

Data from interviews, informal conversations with ANC clients, and observations conducted across eight health facilities reveal the procedural practices, challenges, and complexities surrounding the administration of sulfadoxine–pyrimethamine (SP). Across all data sources, SP administration was largely standardized and conducted under direct supervision by healthcare providers.

The majority of participants reported that they were instructed to bring water to the ANC clinic. SP was dispensed by nurses or midwives and consumed on-site, in their presence. Observations across facilities confirmed that women were generally required to take the medication under direct observation, with limited opportunity to take it home. The mode of consumption varied: some women were instructed to chew the tablets before swallowing, while others swallowed them whole.

“*They* [health providers] *ask us to bring water*,* so when they put it [SP] in your palm then you have to chew it and swallow it in their presence*.” (VRCommunity02, IDI004).

“*You drink it there. They don’t allow you to take it* [SP] *outside*.” (ASCommunity03, IDI001).

Several participants described this practice as compulsory, noting that they were required to take SP before being allowed to leave the clinic.“*So*,* they will compel you to take it before leaving; they first ask whether you have eaten. If you have not eaten they will tell you to go and eat and come back to take i*t [SP].” (VRCommunity03, IDI008).

A consistent theme across interviews and observations was the emphasis placed on food intake prior to SP administration. Women were routinely asked whether they had eaten before receiving the drug. Those who had not eaten were advised to eat and return before taking SP. In some facilities, particularly in the Volta Region, healthcare providers further inquired about the type of food consumed. Women who reported having taken light meals such as tea or maize porridge were instructed to eat heavier food before SP administration. “*They [nurses] asked me if I had eaten*,* and I said no*,* so they [nurses] asked me to go and eat*,* and come after*,* which I was given the SP. I just swallowed it like any other medicine. I took it in front of them*.” (VRCommunity01, IDI007).

Conversations with ANC clients corroborated these procedural accounts. For instance, Danaa Ashitey, who was seven months pregnant, reported having taken SP three times, each under direct observation by midwives, with water provided at the facility. While she recalled being told that SP protects both her and the unborn child, she was unable to specify the illnesses the drug prevents.“*She told me that she took it under direct observation… the nurses told her the drug protects the unborn child and her*,* but she couldn’t tell the kind of ailments it protects them from*.” (EJGovHospital002, conversation with an ANC client).

### Post-SP advice and counseling by health providers

A small number of participants reported receiving advice following SP intake. This included recommendations to avoid prolonged sitting outdoors in the evening, drink plenty of water, rest or sleep if feeling weak, avoid taking folic acid immediately after SP, and consume fruits or toffees to reduce the likelihood of nausea or vomiting.

“*They told me not to sit outside in the evening for too long*.” (ASCommunity04, IDI001).

*“They tell us to take fruits after taking the drug*.” (ASCommunity04, IDI006)

“They tell us once you have taken the drug, you will sometimes become weak, so when you go home, sleep for a while.” (ASCommunity04, IDI010).

Interview data, conversations, and observations reveal that post-SP counseling was rarely done and inconsistent. The majority of participants reported receiving little or no information after taking SP.

“*They didn’t tell me anything after drinking the medicine*.” (ASCommunity04, IDI002).

“*They don’t say anything to us after we have taken the SP*.” (VRCommunity01, IDI001).

Direct observation further confirmed this pattern. At one facility, Yenpoka Aberiga, a 16-year-old primigravida at approximately five months’ gestation, was administered SP after being asked whether she had eaten. She took the drug with sachet water and was immediately asked to make way for another client(ASFacility03, observation, FN012). In a subsequent conversation, she stated that the nurse had not explained the purpose of the SP, consistent with what was observed during the encounter (ASFacility03, conversation with an ANC client). Similarly, Edna, who reported having taken SP four times at Ejisu Government Hospital, stated that she did not receive any explanation before or after SP administration during her ANC visits (ASFacility02, conversation with an ANC client).

### Knowledge of the benefits of intermittent preventive therapy in pregnancy

Findings from in-depth interviews, informal conversations with antenatal care (ANC) clients, and observation notes indicate that many pregnant women understood sulfadoxine–pyrimethamine (SP) primarily as a preventive medication against malaria for both the mother and the unborn child. Nearly half of the participants across data sources consistently emphasized SP’s role in malaria prevention, often describing it as a drug that protects the pregnant woman and prevents transmission of malaria to the fetus.

“*That one [SP]*,* they say it is a malaria drug*,* so when you take it*,* it helps in protecting you and the baby from malaria*.” (ASCommunity01, IDI004).

“*It protects you and the child from malaria. The mother can pass on the malaria to the baby if she has it. So*,* if you drink the medicine*,* it cures the malaria*.” (ASCommunity04, IDI003).

“*When asked if Adiza knew about the benefits of the SP to a pregnant woman*,* she responded that it helps to protect both the pregnant woman and the unborn child from malaria*.” (ASFacility01, conversation with an ANC client).

Beyond malaria prevention, some participants highlighted what they perceived as broader benefits to the unborn child, including ensuring that the baby remains healthy and strong. “*It helps the unborn child not to get the disease and also makes the child strong*.” (VRCommunity02, IDI010).

The majority of participants reported that information about the benefits of SP was usually provided before the medication was administered at ANC clinics. In addition to explaining SP’s malaria-preventive role, healthcare providers reportedly asked women about their use of insecticide-treated bed nets and encouraged continued use. Observations at health facilities further confirmed that SP was commonly administered directly at the clinic under supervision, with women rarely allowed to take the medication home.

Some women reinforced their understanding of SP’s effectiveness through personal experiences, particularly when laboratory tests showed no malaria parasites after repeated SP intake.

“…*it prevents me from getting malaria. Whenever I go to the laboratory*,* they do not find malaria parasites in my blood*.” (ASCommunity04, IDI009).

“*The malaria drug is to prevent malaria. It prevents malaria so that you do not get malaria*,* because you may have had the mosquito bites already*.”(ASCommunity03, IDI001).

However, triangulation of interviews, conversations, and observations also revealed misconceptions about the scope and function of SP. A few participants believed that SP provided complete protection against malaria, thereby eliminating the need for other preventive measures such as sleeping under mosquito nets or avoiding mosquito bites.

“*It helps because I can stay outside very late and I do not also sleep in a net. Without that*,* I would always be going to the hospital for malaria treatment*.” (ASCommunity03, IDI001).

“*The midwife told me the drug will protect me from malaria and that is the main reason I do not even sleep in the net… I am already protected*,* so I do not see the need to sleep in the mosquito net.*” (ASFacility02, conversation with an ANC Client).

Other misconceptions extended beyond malaria prevention. Some women attributed additional protective effects to SP, such as preventing rashes in newborns or ensuring that a baby’s eyes open normally after birth. “*The ‘3–3’ [SP] is good. It protects the baby from malaria… when you deliver the baby*,* rashes may appear on the body*,* or maybe another child’s eyes too might not open*,* so it prevents them from the afflictions that I mentioned*.” (ASCommunity01, IDI005).

### Frequency of SP uptake and knowledge of recommended dosing

Findings from interviews and facility observations reveal considerable variation in both the reported frequency of SP uptake among pregnant women attending antenatal care (ANC) and their understanding of how often the drug should be taken during pregnancy. While most women confirmed that they received SP during ANC visits, there was substantial inconsistency in the number of doses taken and limited awareness of the recommended dosing schedule.

Across interviews, participants reported receiving between two and five doses of SP during their pregnancies. Some women indicated that they had completed SP intake after three doses, while others reported continued administration up to the eighth month of pregnancy.

“No, I am done taking it. They said you have to take it three times from the 4th month to the 7th month. I am in my 8th month now, so I am done.” (VRCommunity02, IDI002).

“*I started taking it in my fifth month. I think I have taken it five times.*” (ASCommunity03, IDI008).

With regard to frequency of administration, several participants reported that SP was given at every ANC visit, while others stated that it was administered monthly.

“*Whenever I go to the hospital*,* they give me some. I have been given six times*.” (ASCommunity04, IDI009).

“*I take it every month*,* and when you are close to delivery*,* you report every two weeks. So*,* I have taken it many times*.” (ASCommunity03, IDI007).

Some women expressed uncertainty about whether the number of doses they had received aligned with what was recommended, indicating confusion and a lack of clear guidance from healthcare providers. “*They said three times*,* but I have been given four*,* so I have decided that if they give me some again in any of my next visits*,* I will ask them*.” (ASCommunity03, IDI001).

Participants with prior pregnancy experience noted changes in SP administration over time. These women reported that while they had previously received three doses in earlier pregnancies, SP was now administered more frequently. “*At first we were taking it three times*,* but now any time you go there they will give you some*.” (ASCommunity03, IDI009).

When asked whether they had been informed about the number of times SP should be taken during pregnancy, most participants mentioned being told that SP should be taken three times, although a few reported being told four times.

“*They told us we will take it three times*.” (ASCommunity04, IDI010).

“*They told me I will take it four times.*” (VRCommunity04, IDI006).

However, a significant number of participants stated that they had not been informed about the recommended number of doses. Instead, they relied on healthcare providers’ instructions and took SP whenever it was administered during ANC visits.

“*I do not know why they let us take it that much*,* but when you are close to delivery*,* they let you stop taking it*.” (ASCommunity03, IDI008).

“*I do not know*,* but the nurses know the number of times that I am supposed to take it [SP]. So anytime they give me*,* I take it.*” (VRCommunity02, IDI001).

“*I don’t know that [number of times she is required to take SP]*,* but I was asked to take and I took it*.” (VRCommunity04, IDI001).

Observational data corroborated these accounts. For instance, during a review of Adiza’s Maternal Record Book, it was observed that she had received her first SP dose at a previous ANC visit and her second dose at the time of observation. When asked whether she knew how many times she was supposed to take SP, she expressed complete reliance on healthcare providers: “*If they* [midwives] *give it* [SP] *to me*,* I will take it*,* but if they [midwives] do not give it to me*,* I cannot say anything.*” (ASFacility01, conversation with an ANC client).

Further interviews revealed that some women held incorrect or incomplete knowledge about SP dosing, including misconceptions about timing, number of tablets per dose, and total number of doses required. Some participants believed that receiving three tablets at once was linked to missed ANC visits, while others perceived that SP is only administered from six months of gestation.

“*It depends on the gestational age. So*,* if you do not attend ANC for about three months*,* when you go they will give you three [SP tablets] for you to take them all. But if you attend ANC every month*,* then you would be given only one [*SP] *tablet*.” (ASCommunity01, IDI008).

“…*we are also given some three white tablets to chew in their presence when the pregnancy gets to about six months.*” (VRCommunity02, IDI007).

### Experience in taking in SP

Many study participants shared their experiences in taking SP, such as not liking the smell, the drug tasting bitter, feeling nausea, or feeling weak after intake. Others expressed concerns or discomfort associated with SP intake, such as stomach aches, dizziness, and vomiting after taking SP. Also, it was reported that pregnancy can make it difficult for some to eat, which presents a challenge as the health providers demand that pregnant women should eat before taking in SP.

*“The malaria drug*,* when it comes*,* it is bitter*,* extremely bitter. If you are not determined*,* you will not take it. If you do not take it in the presence of the midwives/nurses*,* they will not give it [SP] to you to take it home*,* so they will cease your card and make you buy water to take it.”*(ASCommunity01, IDI005).

*“…there are some medicines we drink at the hospital… they gave me a medicine*,* which is 3 in number*,* and I chewed it there. I came home after taking the medicine*,* I felt weak*,* but after some time I regained my strength.”* (ASCommunity04, IDI002).

*“We vomit whenever we take it.”* (ASCommunity04, IDI006).

*“Yes*,* medicine for malaria*,* and you drink it at the hospital. They will not allow you to bring it home because it is very difficult to drink it. I nearly vomit after drinking it [laughing].”* (ASCommunity04, IDI003).

*“Ehe! The malaria one; Oh! The last time I took it I could not do any work. My whole system felt disturbed; my stomach ached*,* and the child also struggled moving in different directions. I think it was only today*,* which is three days after*,* that I am feeling fine.”* (VRCommunity03, IDI004).

*“For me*,* when I take the SP*,* I feel like vomiting*,* and it makes me dizzy and also makes me eat more.”*( VRCommunity04, IDI 008).

A few of the study participants reported no adverse effects when taking SP, although some acknowledged being aware that others had experienced adverse outcomes.

*“As for me*,* when I take it*,* I do not feel anything.”* (ASCommunity01, IDI003).

*“Personally*,* I do not feel anything.”* (ASCommunity01, IDI010)

“No, I do not get any problem, but some people become weak and vomit. When I take it, I do not experience anything.” (ASCommunity04, IDI007).

“Please, I do not have any problem with taking drugs whilst pregnant. I don’t vomit or have any problem when I take drugs. I haven’t experienced that in my life.” (ASCommunity04, ID1004).

### Intentions for future uptake of SP

Data from in-depth interviews, informal conversations, and facility observations indicate that the majority of study participants intended to take SP in future pregnancies. This intention was most commonly linked to women’s perceptions of the protective benefits of SP, particularly its role in preventing malaria and safeguarding the health of both the mother and the unborn child. Many participants expressed a willingness to comply with the recommended SP dosage, even when they described the medication as unpleasant or difficult to take.

Several women framed future SP uptake as unavoidable, because of the perception that it is compulsory, and also emphasized that the perceived health benefits outweighed the discomfort associated with the drug.

“*I do not have any option. I have to drink it [SP] because if you have malaria*,* you can pass it on to the baby*,* though it is very difficult to drink it.”* (ASCommunity04, IDI003).

“*When I become pregnant again*,* I will like to take it again*.” (ASCommunity04, IDI009)

“I will like to take it again, because when I take it, it prevents me from those parasites.” (ASCommunity04, IDI006).

“*Oh*,* why not! For the sake of my health*,* I will take it*.” (ASCommunity03, IDI008).

Observational and conversational data further reinforced these findings. For instance, Madam Grace, a nine-month-pregnant woman interviewed at a government hospital, reported experiencing pronounced weakness whenever she took SP and indicated that she would have preferred an alternative if one were available. Despite this, when asked whether she would take SP in a future pregnancy, she laughed and confirmed that she would, highlighting the tension between negative bodily experiences and strong beliefs in the drug’s benefits (ASFacility02, FN013, conversation with an ANC client).

However, a minority of participants expressed uncertainty or reluctance regarding future SP uptake. These concerns were largely shaped by the drug’s smell, taste, physical discomfort, and previous adverse reactions. Some women described their willingness to take SP as contingent on how their bodies responded in subsequent pregnancies, while others expressed a firm intention not to take it again.

“*You know every pregnancy has its own way it makes you feel. If my next pregnancy permits me to take it*,* I will take it*,* but for now I have decided not to take it again.*” (ASCommunity03, IDI011).

“*I started taking it the first month I started*,* and I am currently seven months. I take it every time I go there. In fact*,* that drug I won’t take it again*.” (ASCommunity03, IDI011).

## Discussion

This ethnographic study explored pregnant women’s knowledge, attitudes, and practices regarding the use of SP during antenatal care (ANC) in two regions of Ghana. Guided by the Health Belief Model (HBM), the analysis focused on three core constructs: perceived susceptibility, perceived seriousness, and perceived benefits and barriers [18] to understand factors influencing SP uptake. Major themes identified included the context of SP administration as observed at the ANC clinics, Post-SP advice and counseling by health providers, knowledge of the benefits of intermittent preventive therapy in pregnancy (IPTp-SP), frequency of SP uptake and knowledge of recommended dosing, experiences in taking SP, and intentions for future uptake of SP.

This study found that most participants received three or more doses of SP during pregnancy, largely under directly observed therapy (DOT). The high uptake observed can be attributed to the strict enforcement of DOT across the eight health facilities, consistent with Ghana National Malaria Elimination Programme recommendations [32, 33]. This finding contrasts with earlier studies in Ghana that reported lower uptake, with fewer than half of postpartum women receiving at least three doses of SP [17], and only two doses reported among most pregnant women in the Atwima Kwanwoma District [34]. Evidence from reviews conducted in 2022 and 2024 supports the finding that DOT facilitates SP uptake [12, 35]. Conversely, studies from Nigeria have shown that poor supervision contributes to low uptake [36]. However, while DOT was effective in ensuring high uptake in this study, it also limited women’s autonomy in decision-making regarding SP. Despite this, women’s perceived susceptibility to malaria during pregnancy appeared to motivate acceptance of SP, aligning with earlier studies that link higher IPTp-SP uptake to perceived severity of malaria in pregnancy [37, 38]. Similarly, perceived susceptibility to pregnancy-related complications has been associated with increased ANC attendance [21].

The majority of participants recognised the importance of SP in preventing malaria during pregnancy, demonstrating a general understanding of how IPTp-SP works. This perception strongly influenced uptake and aligns with the HBM’s assertion that perceived benefits encourage health-promoting behaviours. Comparable findings have been reported in studies applying the HBM to other contexts, such as maternal COVID-19 preventive behaviours [15, 39]. Participants’ relatively good knowledge of SP benefits is unsurprising, as interviews, conversations, and observations revealed that most women were informed about SP’s preventive role before their first dose. This finding aligns with previous studies in Ghana reporting good knowledge of IPTp-SP among pregnant women [14, 40–42]. Other studies have similarly found that knowledge of SP benefits positively influences uptake [35–37, 43], although Ansong et al. [15] reported that low knowledge of benefits contributed to poor uptake.

Despite general awareness of SP’s benefits, a substantial number of women expressed misconceptions about its purpose and scope of protection. Some believed SP provided complete protection against malaria, eliminating the need for additional preventive measures such as sleeping under insecticide-treated nets. This was observed despite health workers providing information on SP and distributing free long-lasting insecticide-treated nets (LLINs). These misconceptions may be partly explained by low literacy levels among participants. Previous studies have identified low literacy as a barrier to intervention uptake [14, 44], while literate women are more likely to understand health information and perceive their susceptibility to malaria [14, 35]. Literacy has also been shown to empower women to access information, exercise autonomy, and utilise maternal health services, including IPTp-SP [45–47].

Women in their second or subsequent pregnancies noted an increase in SP dosage from three doses to four or more, but were largely unaware of the reasons for this change and rarely sought clarification at ANC clinics. This reflects the implementation of Ghana’s revised policy, which recommends monthly SP administration from the second trimester until delivery [8]. Similar findings have been reported in Ghana, where most women now receive more than three doses of SP [15, 38, 40]. Evidence from Kenya and Tanzania suggests that increased SP dosing improves maternal outcomes, although gaps in patient–provider communication about policy changes persist [48, 49]. In contrast, a study in Uganda reported improved compliance when healthcare workers proactively explained dosage changes during ANC [50]. In this study, acceptance of increased dosing among multiparous women may have been influenced by perceived benefits based on prior pregnancy experiences, consistent with earlier findings [21].

Participants commonly reported side effects such as nausea, dizziness, and vomiting, particularly after initial SP doses. These experiences served as perceived barriers to uptake, consistent with WHO reports and other studies documenting similar adverse effects [3, 36]. While side effects are often temporary and diminish with subsequent doses [51], they can discourage adherence if not adequately addressed. Studies have shown that prior counseling about SP side effects and benefits can improve adherence, particularly among younger mothers [52]. Health workers’ practices, such as asking about food intake and advising women to eat before taking SP, suggest an awareness of these adverse effects. However, the lack of water provision in some facilities emerged as a structural barrier to effective DOT. Similar barriers, including shortages of water and cups, have been reported elsewhere [37, 53].

Although SP uptake was widespread, most women lacked accurate knowledge of the recommended dosing schedule and relied heavily on healthcare providers for guidance. This communication gap mirrors findings from Ghana showing that insufficient provider guidance leads to incomplete IPTp-SP regimens [14, 16, 40–42]. In contrast, women who received education on SP timing and follow-up doses were more likely to complete the recommended regimen [34, 42]. Lack of dosing knowledge has been identified as a perceived barrier to uptake in other studies [17]. The integrated ANC model observed across facilities may partly explain these gaps. While integrated care provides comprehensive services, including malaria prevention, HIV testing, nutrition counseling, and LLIN distribution, it may limit time available for detailed SP counseling. Nevertheless, integrated ANC has been shown to facilitate service uptake and improve provider–client interactions, which can enhance SP adherence [16, 23, 53].

All the antenatal care (ANC) facilities observed operated an integrated ANC system, ensuring that women received comprehensive maternal healthcare services, including malaria prevention in pregnancy and HIV testing. Counselling services, nutrition education, and the distribution of long-lasting insecticide-treated nets (LLINs) to newly registered ANC clients were also routinely provided. Given that health workers had already provided information before administering SP, they may have felt less need to repeat explanations at the point of uptake and instead focused on delivering other essential services. Previous studies have highlighted the importance of integrated service delivery, noting that it enhances provider–client interaction and can generate psychological benefits that contribute to increased SP uptake [16, 23]. Health provider-client interation can improve interactional quality such as positive provider attitudes, empathy, and compassion, which has also been identified as an important facilitator of SP uptake [21, 54–56]. In Ghana, the integrated ANC approach requires providers to deliver multiple services within a limited timeframe. After counselling women and administering SP, health workers typically proceeded to provide other ANC services. This integrated model is perceived to facilitate SP uptake because it encourages women to attend ANC regularly in order to access a range of services. Regular attendance, in turn, creates opportunities for consistent SP administration and higher overall uptake, as reported in other studies on SP coverage [12, 53], as well as in integrated service models for interventions such as HIV testing [57].

Most women indicated willingness to take SP in future pregnancies, particularly if provided with adequate information about its relevance. Intentions for future uptake were driven primarily by perceived benefits, even in the presence of side effects, supporting a central tenet of the HBM [2, 3]. Trust in healthcare providers further reinforced adherence, consistent with findings from Cameroon and broader literature on patient trust and compliance [58–62]. However, women who experienced adverse effects were less likely to achieve optimal dosing in some studies [44]. The findings also highlight power dynamics within ANC settings, where women often deferred to provider authority without questioning treatment decisions. This reflects broader societal norms and the paternalistic nature of healthcare delivery in Ghana, as noted in previous studies [63, 64].

### Study limitations

As a qualitative study, the findings are not generalisable beyond the study context due to purposive sampling and a limited number of participants. Few pregnant women not attending ANC were identified, constraining the analysis of non-uptake of SP. Additionally, reports from women allowed to take SP home were self-reported and could not be independently verified. Also, translating in-depth interviews and conversations from Ewe and Twi into English may have caused slight loss of meaning or cultural expressions, even though bilingual transcription and review were used to improve accuracy. Nonetheless, the findings are consistent with existing literature and provide valuable insights into contextual factors influencing IPTp-SP uptake in Ghana.

## Conclusion

The findings of the study suggest a need for consistent communication and education on SP, it’s benefits in malaria prevention, required doses, and the side effects. Considering that most of the women who utilized the services did not have tertiary education, and their main source of information was the health providers.

Healthcare providers should focus more on patient-centered communication, consistent counseling, and guidance on the management of side effects to support sustained adherence.

Also, misconcpetions were noted regarding it being a stand alone intervention, it is important that women are made to understand that SP is part of a package of interventions, which includes the use of LLINS to prevent malaria in pregnancy and testing and treating when there is the suspicion of malaria in pregnancy.

## Supplementary Information

Supplementary Material 1.

Supplementary Material 2.

Supplementary Material 3.

## Data Availability

Data is available at the University of Health Research repository and can be made available upon reasonable request from the first author, Matilda Aberese-Ako (MA).
