# Supplementary material for: Knowledge, attitudes, practices, and future intentions to use intermittent preventive treatment with sulfadoxine-pyrimethamine among pregnant women in southern Ghana
Source: BMC Pregnancy Childbirth. 2026 Apr 15;26:562. doi: 10.1186/s12884-026-09063-8 (PMC13191985; doi:10.1186/s12884-026-09063-8)
Supplement: Supplementary file 1 — Supplementary Material 1. [file 12884_2026_9063_MOESM1_ESM.docx]

**IDI GUIDE FOR STUDY ON ‘Interpersonal, socio-cultural, environmental and community factors determining pregnant women’s decision to control malaria infection in Ghana: An anthropological study’**

**Introduction**

This is part of a larger study, which seeks to ascertain the burden of prenatal parasitic infections and the factors, including individual and community level factors, which contribute to the prevalence of maternal anaemia and incidence of LBW among a cohort of pregnant women attending antenatal care clinics in district hospitals in the Ashanti and Volta regions of Ghana. This qualitative aspect of the study seeks to understand the interpersonal, community, environmental and socio-cultural factors that influence pregnant women’s decisions to control malaria **and other parasitic** infections. The study will also explore how these factors individually and or collectively influence pregnant women’s decisions to control malaria infections. Recommendations and suggestions on how to improve malaria control among households and pregnant women will be explored. You are being invited to join this study because you have been identified as a pregnant woman/a woman who gave birth in the last twelve months. Your honest responses would be very helpful as it would contribute to understanding how community factors influence decision to control malaria infection and the possible recommendations to improve interventions aimed at controlling malaria infection among pregnant women in Ghana.

**Section 1: Background characteristics**

Name

Age

Location

Level of education of woman

Level of education of partner

Occupation of woman

Occupation of partner

Type of housing

Number of occupants per room

**Section 2: Interpersonal issues that influence knowledge, attitudes and health seeking behaviour**

- What do you know about Malaria?
- Why is Malaria an important issue for pregnant women?
- How does malaria affect pregnant women?
- Tell me about your experiences in preventing malaria since you became pregnant?
  - What about treating malaria since you became pregnant?
- Prior to your current pregnancy how were you protecting yourself against malaria infection?
- Have you had other previous pregnancies, if yes, how did you control malaria infections during your previous pregnancies?

**Section 3: Community related factors influencing control of malaria** **infection**

- Do you have health education programmes in this community?
- What kinds of associations do you have in this community?
  - Do you belong to any association?
- Do you discuss any issues on health care?
- What about issues on malaria?
  - If yes, what are the issues?
- How has it improved your knowledge on malaria infection in pregnant women?

**Socio-cultural factors that influence attitudes and practices in controlling malaria infections**

Household dynamics and decision making

- When a member of your household is taken ill, what is usually done?
- When a member of the household is sick who decides where the fellow should go to?
- What about the pregnant woman, who decides where she should go for health care?
- Who is responsible for paying the cost of health care?
- What is/are the role(s) of the following people in decisions on the pregnant woman accessing healthcare?
  - Household head, husband, mother-in-law
- How much are you willing to spend on buying drugs or a bednet to protect against malaria infection during pregnancy? Who pays for it?
- How much do you pay?

**Traditional system**

- Are there any taboos relating to pregnancy?
  - What are they?
- Which are the traditional places or avenues that a sick pregnant woman can visit for health care?
- Please tell me about your experiences in using the traditional system of health care?
- What are the cultural practices relating to the first visit to the ANC clinic?

**Environmental factors influencing malaria infection**

- Kindly tell me about your visits to the hospital, when you become pregnant?
  - What do you go there for?
- How far is the hospital from your home?
- When is it appropriate for the pregnant woman to visit health facility for ANC?
- Do you share a room with others?
- How do you protect yourself from mosquito bites at night?
- What do you know about bed nets, can you tie one?
- Do you use one a bed net when you go to bed at night? (if yes/no probe why for the specific response)
- How often do you use it?
  - When during the year do you use bed nets?
  - Probe why that particular season?
  - Why do you use it?

**Attitudes and health seeking behaviour that influences SP access**

- What stage of pregnancy do you think is best to visit the hospital or to start discussing with the health worker on how to protect yourself from malaria and other infectious diseases?
- What informs your decision to use health facilities?
- What informs your decision to treat malaria?
- Do you attend ANC?
  - At what stage of the pregnancy did you begin to attend ANC?
- What factors do- you consider before making your first ANC visit?
- What were you offered at the ANC clinic?
- How many times have you visited the ANC clinic during this current pregnancy?

**Attitudes towards malaria infection, treatment and health care interventions**

**Alternate solutions**

- What is the best way to control malaria?
- What do you think is the best strategy to reach communities on malaria education?
- What are the possible ways to reach women who are not yet pregnant?
- What about other community members?

**Knowledge and attitude towards other parasitic infections**

- Do you know of other infections besides malaria that can affect you and your unborn baby? Please mention them.
- How are they acquired? Probe for each parasitic infection mentioned
- How are they be prevented?
- How can they be treated? Probe for health facility treatment, traditional remedy, spiritual etc.
- How can you prevent yourself and your unborn child from acquiring such infections?

Thank you for agreeing to this interview
