# Supplementary material for: Knowledge, attitudes, practices, and future intentions to use intermittent preventive treatment with sulfadoxine-pyrimethamine among pregnant women in southern Ghana
Source: BMC Pregnancy Childbirth. 2026 Apr 15;26:562. doi: 10.1186/s12884-026-09063-8 (PMC13191985; doi:10.1186/s12884-026-09063-8)
Supplement: Supplementary file 2 — Supplementary Material 2. [file 12884_2026_9063_MOESM2_ESM.docx]

Supplementary Observation list 1

Date: 24^th^ July, 2018

**Bednet**

Observe how it is given out to pregnant women.

The message the pregnant women are given on how to use it.

**IPTp**

Observe ANC consultation. How nurse provides ANC services

How SP given

What message is the pregnant woman given before being given SP?

What message is the pregnant woman given after taking the SP?

**Malaria cases**

Observe how women with malaria present at the facility.

Observe how the nurse goes about the management.

Whether woman is treated or asked to go for test or undergoes RDT

After RDT or lab test what happens: how is treatment given, what is pregnant woman told by nurse?

How is the pregnant woman treated at the lab?

How is the pregnant woman treated at the pharmacy?

**Admission/detained**

Where women are detained or admitted for malaria, find out at what stage that women are admitted

If women are able to hold a discussion find out how they got malaria

If unable to talk take their contact and after they are discharged find out how they got malaria. But note those allergic to SP.

Table for observation

Consulting room no.

Date:

| Client no. | IPTp given under DOT | Given without DOT | IPTp Not given | Comment (Education given or not before and after IPTp) |
| --- | --- | --- | --- | --- |
|  |  |  |  |  |
|  |  |  |  |  |
|  |  |  |  |  |
|  |  |  |  |  |
|  |  |  |  |  |
|  |  |  |  |  |
|  |  |  |  |  |
|  |  |  |  |  |

Bednet

| Client no. | Given bednet | Comment (Education given or not before and after issuance) |
| --- | --- | --- |
|  |  |  |
|  |  |  |
|  |  |  |
|  |  |  |
|  |  |  |
|  |  |  |
|  |  |  |
|  |  |  |

Malaria treatment

| Client no. | Test conducted | How treatment is provided | Comment (interaction with nurse, education given etc) |
| --- | --- | --- | --- |
|  |  |  |  |
|  |  |  |  |
|  |  |  |  |
|  |  |  |  |
|  |  |  |  |
|  |  |  |  |
|  |  |  |  |
|  |  |  |  |
